# Determinants of Malaria Vaccine Awareness Among Women in Ghana: Findings From the 2022 Ghana Malaria Indicator Survey

**DOI:** 10.1002/puh2.70126

**Published:** 2025-09-26

**Authors:** Michael Sarfo, Augustina Konadu Larbi‐Ampofo, Olanrewaju Lawal, Sandra Konadu Bonnah, Gilbert Eshun

**Affiliations:** ^1^ School of Human and Health Sciences University of Huddersfield Huddersfield UK; ^2^ Coventry University Coventry UK; ^3^ Acute Medicine Unit Epsom and Saint Helier University Hospitals Carshalton UK; ^4^ Department of Health Promotion and Disease Prevention University of Nebraska Medical Center Omaha Nebraska USA; ^5^ Paediatric Department Kwame Nkrumah University of Science and Technology Hospital Kumasi Ghana; ^6^ Seventh Day Adventist Hospital Agona‐Asamang Ghana; ^7^ The Royal (Dick) School of Veterinary Studies and the Roslin Institute University of Edinburgh Midlothian UK

**Keywords:** awareness | Ghana | malaria vaccine | women

## Abstract

**Background:**

In Ghana, pregnant women are disproportionately affected by malaria, yet awareness of malaria vaccination remains limited among women of reproductive age. This study examines sociodemographic determinants of malaria vaccine awareness among Ghanaian women using data from the 2022 Ghana Malaria Indicator Survey (MIS).

**Methods:**

We analysed data from 38,459 women aged 15–49 years. Awareness was defined as having heard of the malaria vaccine within the past 6 months. Descriptive statistics and logistic regression were used to assess associations between awareness and sociodemographic characteristics. The analysis accounted for survey weights, clustering, and stratification. Adjusted odds ratios (AORs) with 95% confidence intervals (CIs) are reported, with clearly defined reference groups.

**Results:**

Overall, awareness of the malaria vaccine was higher among women who had tertiary education (AOR = 2.89, 95% CI: 2.50–3.34, ref: no education), used the internet in the past 12 months (AOR = 1.85, 95% CI: 1.74–1.97, ref: no internet use), and resided in Greater Accra (AOR = 1.75, 95% CI: 1.54–2.00, ref: Western region). Compared to adolescents aged 15–19 years, awareness increased with age, peaking among women aged 45–49 (AOR = 2.03, 95% CI: 1.60–2.57). Religious affiliation and ethnicity were also associated with awareness, though to a lesser extent.

**Conclusion:**

Malaria vaccine awareness among Ghanaian women is shaped by access to education, digital connectivity, and regional and religious contexts. Public health campaigns should prioritise equitable access to vaccine information, especially for underserved populations, to support Ghana's 2028 malaria elimination goals.

## Graphical abstract

Malaria vaccine awareness in Ghana is driven by education, digital access, age, religion, and region. Closing digital and regional gaps is essential to reach underserved women and support Ghana's malaria elimination goals.

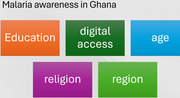

## 1 Introduction

Malaria is still a major public health issue, responsible for a significant proportion of global morbidity and mortality, especially in sub‐Saharan Africa [1]. According to Bediako et al. [2], malaria disproportionately affects low‐income regions and poses severe health risks, particularly to children and women in endemic countries. In Ghana, malaria is an endemic disease that threatens the entire population [3]. The World Health Organisation (WHO) 2021 report states that Ghana accounted for 2.1% of global malaria cases and 1.9% of malaria‐related fatalities in 2020 [4]. In 2017, malaria accounted for 40% of all outpatient visits to hospitals in Ghana [5]. Additionally, Ghana was among the top 11 countries with the highest malaria burden in 2018, contributing 70% of estimated global cases and 71% of estimated deaths worldwide [6].

Although Ghana has made substantial progress in reducing malaria mortality and is currently classified as being in the ‘control’ phase, the prevalence of malaria‐related illness and death remains a major concern [5]. Ghana is also one of the 15 countries with the highest malaria hotspot index, contributing around 3% of global malaria morbidity [7]. Despite being preventable and treatable, malaria continues to have a significant negative impact on public health, especially among women and children in developing countries [8, 9]. The burden of malaria infection is disproportionately borne by vulnerable populations [10]. According to WHO reports from 2019 and 2022, malaria remains most prevalent among infants, pregnant women, travellers, and individuals living with HIV/AIDS [11]. A study by Bardoe et al. [12] found a malaria prevalence of 10.8% among pregnant women, higher than the national prevalence of 8.6%, whereas research studies in Ghana reported a 14.1% and 15.2% prevalence among pregnant women in the Northern region [13, 14].

To reduce healthcare costs and improve service utilisation, especially among poor women of reproductive age, Ghana introduced the National Health Insurance Scheme (NHIS) in 2005 [15]. Individuals aware that malaria treatment is covered by the NHIS are less likely to self‐medicate and more likely to seek treatment at healthcare facilities [16]. Furthermore, awareness of NHIS coverage has been associated with increased uptake of intermittent preventive treatment during pregnancy (IpTP) [17]. Although NHIS enrolment is expected to encourage formal healthcare‐seeking behaviour, the high number of malaria cases raises questions about accessibility and public awareness of NHIS benefits [18].

Vaccination is one of the most effective tools in public health for preventing disease in both children and adults, particularly when effectively communicated and accepted by communities [19]. The development of a malaria vaccine has been a long‐standing challenge due to the complex life cycle and antigenic variation of the Plasmodium parasite [20]. Despite these difficulties, significant advances have been made [21]. The RTS, S/AS01 and R21/Matrix‐M vaccines are two important milestones. The WHO has recommended their use in areas with moderate‐to‐high *Plasmodium falciparum* transmission, especially in sub‐Saharan Africa, based on strong evidence of their protective efficacy from large clinical trials [22].

Following a successful pilot programme by GSK, PATH, the Ministry of Health, Ghana, Gavi, the Vaccine Alliance, the Global Fund, and WHO, the malaria vaccine has been introduced into routine immunisation services in Ghana [23]. Community acceptance is crucial for successful vaccine implementation [21]. A recent review found high levels of acceptance for the RTS, S malaria vaccine in low‐ and middle‐income countries (LMICs), with an average rate of 95.3% [24]. However, acceptance rates vary and are influenced by sociodemographic factors and community concerns about safety, effectiveness and awareness [25, 26].

One key to successful implementation and uptake of vaccines is awareness, especially among women of reproductive age. The media has traditionally played a central role in promoting public health awareness, particularly when introducing new interventions [27]. This study seeks to address the current gap in knowledge by using secondary data from the Demographic Health Surveys (DHS) to examine the sociodemographic factors influencing awareness of malaria vaccination among women in Ghana.

## 2 Methods

### 2.1 Study Design and Measures

This study employed a cross‐sectional design to examine the association between sociodemographic characteristics and malaria vaccine awareness among Ghanaian women. Awareness was operationalised as having heard of the malaria vaccine.

The primary data source was the 2022 Ghana Malaria Indicator Survey (MIS), specifically the women's dataset. Sociodemographic characteristics considered in the analysis included DHS. These variables were selected on the basis of their availability in the DHS dataset and their relevance in prior literature examining health information access and vaccine awareness [28].

### 2.2 Data Source and Collection

This study utilised secondary data from the 2022 Ghana MIS, a nationally representative household survey conducted by the Ghana Statistical Service (GSS), Ghana Health Service (GHS), and the National Malaria Elimination Programme (NMEP), in collaboration with ICF International under the Demographic Health Surveys (DHS) Program.

The 2022 MIS was designed to generate reliable estimates of malaria‐related indicators at the national, urban–rural, and regional levels. A two‐stage stratified sampling design was employed. In the first stage, enumeration areas (EAs) were selected using probability proportional to size. In the second stage, households within each EA were systematically sampled. All women aged 15–49 years who were either permanent residents or visitors who stayed in the household the night before the survey were eligible for interview.

Data were collected between November 2022 and February 2023 using standardised questionnaires administered through face‐to‐face interviews. Trained enumerators used digital tablets with built‐in data quality controls to ensure accuracy and completeness. The survey covered a wide range of topics, including household characteristics, mosquito net ownership and use, fever management, malaria testing, and malaria prevention during pregnancy. Further information regarding the methodological approach for the MIS data collection can be found on the official site of the DHS programme via: http://goo.gl/ny8T6X.

### 2.3 Variable

#### 2.3.1 Outcome Variable

In the survey, women were asked about ever heard of the malaria vaccine. We categorised the outcome variable as 0 = No, 1 = Yes. The outcome variable, awareness of the malaria vaccine, was operationalised as a binary measure based on whether a respondent had heard of the malaria vaccine in the past 6 months. This variable captures general awareness, irrespective of the depth of understanding, content of the message, or exact source of information. However, potential exposure channels were indirectly explored through variables, such as internet use, which may reflect access to digital sources of health information. No additional questions were asked regarding the specific medium or content of the information received.

### 2.4 Explanatory Variables

We included 7 explanatory variables. They include age (15–19, 20–24, 25–29, 30–34, 35–39, 40–44, 45–49), education (no education, incomplete primary, complete primary, incomplete secondary, complete secondary, higher), religion (Pentecostal Orthodox, Islam, traditionalist, Other Christian, no religion), region (Western, Central, Greater Accra, Volta, Eastern, Ashanti, Western North, Ahafo, Bono, Bono East, Oti, Northern, Savannah, North East, Upper East, Upper West), ethnicity (Akan, Ga/Dangme, Ewe, Guan, Mole‐Dagbani, Grusi, Gurma, Mande, other) and use of internet (yes and no). These variables were selected on the basis of their availability in the DHS dataset and their availability in the literature [28]. Reference categories for the regression models were selected on the basis of their relevance to public health policy and their interpretability. For example, younger age groups (15–19 years), no education and no internet use were used as baselines to highlight disparities relative to populations with typically limited health access or awareness.

### 2.5 Data Analysis

Data analysis was conducted using IBM SPSS Statistics version 29.0.2.0. Descriptive statistics (proportions) were used to explore the distribution of malaria vaccine awareness across sociodemographic groups.

We then conducted binary logistic regression analysis to identify predictors of awareness. Both crude odds ratios (CORs) and adjusted odds ratios (AORs) with 95% confidence intervals (CIs) were reported to allow intuitive interpretation of effect sizes. All models accounted for the DHS complex survey design, incorporating sampling weights, clustering, and stratification to ensure nationally representative estimates. Before conducting regression analysis, diagnostic tests for multicollinearity were performed using the variance inflation factor (VIF) and tolerance index (TI). VIF values above 5 and TI values below 0.20 were considered indicative of multicollinearity [29]. The results of these diagnostics are presented in Table A1 at the appendix.

### 2.6 Ethical Consideration

We used a secondary dataset that is freely available to the public from the DHS programme; therefore, no ethical approval was requested. The dataset is anonymised, and more information regarding its ethical standards is available at http://goo.gl/ny8T6X.

## 3 Results

Table 1 shows the distribution of malaria vaccine awareness by socio‐demographic characteristics. Awareness increased with age up to a point, peaking among those aged 25–29 years (61.5%), after which it generally plateaued. The lowest awareness was among adolescents aged 15–19 years (46.5%). Participants aged 30–39 and 35–39 reported similar awareness levels of 59.1% and 59.4%, respectively. Among the older age groups, awareness slightly declined, with 56.6% of those aged 40–44 and 57.4% of those aged 45–49 reporting being aware.

**TABLE 1:** Distribution of malaria awareness by sociodemographic characteristics.

Variables	Subgroup	Total (*n*)	Aware—yes (*n*)	Aware—no (%)
**Age (years)**	15–19	318	148(46.5%)	170(53.5%)
	20–24	1852	1013(54.7%)	839(45.3%)
	25–29	3613	2221(61.5%)	1392(38.5%)
	30–39	6119	3614(59.1%)	2505(40.9)
	35–39	7531	4477(59.4%)	3054(40.6%)
	40–44	7331	4149(56.6%)	3182(43.4%)
	45–49	6114	3508(57.4%)	2606(42.6%)
**Region**	Western	2470	1376(66.8%)	683(33.3)
	Central	4445	2679(74.2%)	930(25.8%)
	Greater Accra	5072	3127(76.5%)	960(23.5%)
	Volta	1719	773(52.9%)	689(47.1%)
	Eastern	3256	1850(69.9)	798(30.1%)
	Ashanti	7593	3343(53.9%)	2857(46.1%)
	Western North	1096	552(58.4%)	394(41.6%)
	Ahafo	842	520(68.3%)	241(31.7%)
	Bono	1271	625(58.8%)	476(43.2%)
	Bono East	1756	644(40.2%)	957(59.8%)
	Oti	1240	547(48.8%)	575(51.2%)
	Northern	3425	1315(41.1%)	1884(58.9%)
	Savannah	947	466(53.7%)	401(46.3%)
	North East	885	330(38.9%)	519(61.2%)
	Upper East	1554	615(42.6)	828(57.4%)
	Upper West	988	367(39.6%)	556(60.2%)
**Educational level**	No education	11,061	4265(58.4%)	5981(41.6%)
	Incomplete primary	1709	853(56.7%)	652(43.3%)
	Complete primary	5040	2522(57.2%)	1888(42.8%)
	Incomplete secondary	2249	2478(32.4%)	1186(67.6%)
	Complete secondary	13,833	7513(66.9%)	3713(33.1%)
	Tertiary (higher)	2368	1496(82.0%)	328(18.0%)
**Religion**	Pentecostal	15,711	8078(62.0%)	4954(38.0%)
	Orthodox	6975	3746(64.0%)	210(34.0%)
	Islam	7781	3918(55.2%)	3286(44.8%)
	Traditionalist	1253	862(74.8&)	290(25.2%)
	Other Christian	5788	276(35.1%)	511(64.9%)
	No religion	951	2819(57.0%)	2126(33.0%
**Sex of household head**	Male	24,391	11,943(56%)	9377(44.0%)
	Female	14,068	7185(62.2%)	4371(37.8%)
**Ethnicity**	Akan	17,035	9412(67.2%)	4586(32.8%)
	Ga/Dangme	2613	1241(57.5%)	917(42.5%)
	Ewe	4097	2113(61.8%)	1306(38.2%)
	Guan	1234	617(58.3%)	442(41.7%)
	Mole–Dagbani	7317	3408(51.1%)	3263(48.9%)
	Grusi	1372	584(47.3%)	650(52.7%)
	Gurma	3267	1075(36.3%)	1885(63.7%)
	Mande	1233	544(49.0%)	567(51.0%)
	Other	280	135(50.6%)	132(49.4%)
**Use of internet**	Never	26,024	11,949(51.3%)	11,321(48.7%)
	Yes, last 12 months	11,409	6603(75.3%)	2170(24.7%)
	Yes, before last 12 months	1026	575(69.1%)	267(30.9%)

*Note:* Total and subgropu distributions are reported directly from SPSS out; minor discrepancies are due to category coding.

Significant regional variation in awareness was observed. The highest awareness was reported in the Greater Accra (76.5%), Central (74.2%), and Eastern (69.9%) regions. Conversely, the lowest levels of awareness were noted in the North East (38.9%), Upper West (39.6%), Northern (41.1%), and Bono East (40.2%) regions.

Awareness increased with educational attainment. Participants with tertiary education reported the highest level of awareness (82.0%), followed by those with complete secondary education (66.9%). In contrast, individuals with incomplete secondary education had notably low awareness levels (32.4%).

Traditionalists had the highest proportion of awareness (74.8%), followed closely by Orthodox Christians (64.0%) and Pentecostals (62.0%). Muslims had a moderate awareness level (55.2%), whereas individuals identifying as ‘Other Christians’ had the lowest (35.1%)

Awareness was higher in female‐headed households (62.2%) compared to male‐headed households (56.0%).

Among ethnic groups, the Akan reported the highest awareness (67.2%), followed by Ewe (61.8%) and Ga/Dangme (57.5%). The Gurma ethnic group had the lowest awareness (36.3%), followed by Grusi (47.3%) and Mande (49.0%).

Internet use was strongly associated with awareness. Among participants who had used the internet in the last 12 months, 75.3% were aware. This compares to 69.1% of those who had used the internet prior to the last 12 months and only 51.3% of those who had never used the internet.

Table 2 presents the results of the logistic regression analysis examining factors associated with awareness of the malaria vaccine among women of reproductive age in Ghana. Both CORs and AORs with 95% CIs are reported.

**TABLE 2:** Factors influencing awareness of malaria vaccine (crude odds ratio [COR] and adjusted odds ratios).

Variable	Category	COR (95% CI)	AOR (95% CI)	*p* value
**Age (years)**	15–19 (ref.)	1.00	1.00	
	20–24	1.389(1.094–1.763)	1.323(1.030–1.699)	0.29
	25–29	1.835(1.457–2.309)	1.880(1.477–2.393)	<0.001
	30–39	1.659(1.324–2.080)	1.777(1.401–2.253)	<0.001
	35–39	1.686(1.346–2.112)	1.889(1.491–2.393)	<0.001
	40–44	1.500(1.198–1.878)	1.773(1.400–2.246)	<0.001
	45–49	1.549(1.235–1.941)	2.026(1.597–2.570)	<0.001
**Region**	Western (ref.)	1.00	1.00	
	Central	1.431(1.271–1.610)	1.469(1.299–1.660)	<0.001
	Greater Accra	1.619(1.440–1.816)	1.752(1.537–1.997)	<0.001
	Volta	0.557(0.485–0.639)	0.0701(0.594–0.827)	<0.001
	Eastern	1.152(1.018–1.303)	1.397(0.225–1.595)	<0.001
	Ashanti	0.581(0.524–0.645)	0.516(0.462–0.475)	<0.001
	Western North	0.696(0.594–0.815)	0.784(0.666–0.924)	0.04
	Ahafo	1.070(0.895–1.279)	1.240(1.031–1.491)	0.022
	Bono	0.652(0.561–0.757)	0.626(0.535–0.733)	<0.001
	Bono East	0.334(0.292–0.383)	0.446(0.386–0.516)	<0.001
	Oti	0.472(0.407–0.548)	0.977(0.828–1.153)	0.782
	Northern	0.347(0.309–0.389)	0.570(0.495–0.655)	<0.001
	Savannah	0.577(0.491–679)	0.916(0.767–1.095)	0.336
	North East	0.315(0.267–0.372)	0.465(0.388–0.556)	<0.001
	Upper East	0.369(0.321–0.424)	0.448(0.383–0.525)	<0.001
	Upper West	0.328(0.279–0.385)	0.387(0.324–0.462)	<0.001
**Sex of household head**	Male (ref.)	1.00	1.00	
	Female	1.331(1.271–1.394)	1.009(0.958–1.063)	0.732
**Ethnicity**	Akan (ref.)	1.00	1.00	
	Ga/Dangme	0.659(0.601–0.723)	0.440(0.393–0.492)	<0.001
	Ewe	0.788(0.729–0.852)	0.791(0.713–0.878)	<0.001
	Guan	0.679(0.598–0.771)	0.639(0.554–0.737)	<0.001
	Mole‐Dagbani	0.509(0.479–0.540)	0.940(0.859–1.030)	0.184
	Grusi	0.438(0.390–0.492)	0.808(0.703–0.927)	<0.002
	Gurma	0.278(0.256–0.302)	0.639(0.572–0.713)	<0.001
	Mande	0.467(0.413–0.528)	0.580(0.499–0.674)	<0.001
	Other	0.497(0.390–0.634)	0.389(0.298–0.507)	<0.001
**Educational level**	No education (ref.)	1.00	1.00	
	Incomplete primary	1.835(1.645–2.047)	1.390(1.235–1.565)	<0.001
	Complete primary	1.873(1.744–2.012)	1.429(1.318–1.548)	<0.001
	Incomplete secondary	2.93(2.706–3.172)	1.824(1.662–2.002)	<0.001
	Complete secondary	2.837(2.683–2.999)	1.817(1.690–1.953)	<0.001
	Tertiary (higher)	6.402(5.645–7.261)	2.891(2.504–3.336)	<0.001
**Religion**	Pentecostal (ref)	1.00	1.00	
	Orthodox	0.928(0.86.2–0.998)	1.476(1.343–1.622)	<0.001
	Islam	0.407(0.348–0.476)	0.558(0.471–0.662)	<0.001
	Traditionalist	1.340(1.240–1.449)	1.354(1.245–1.472)	<0.001
	Other Christian	1.230(1.151–1.314)	1.091(1.014–1.173)	0.020
	No religion	0.254(0.220–2.93)	0.605(0.515–0.712)	<0.001
**Internet use**	Never (ref.)	1.00	1.00	
	Yes, last 12 months	2.883(2.729–3.045)	1.850(1.735–1.974)	<0.001
	Yes, before last 12 months	2.120(1.826–2.461)	1.739(1.486–2.035)	<0.001

*Note:* Variables entered: Age group, Region, Religion, Sex of household head, Education, Ethnicity, Internet use. Survey design (sample weight, strata and primary sampling units) accounted for in all estimations.

Abbreviations: AOR, adjusted odds ratio; CI, confidence interval; COR, crude odds ration; Ref, reference group.

After adjusting for covariates, several factors remained significantly associated with higher odds of awareness. Women who used the internet in the past 12 months were more than twice as likely to be aware of the malaria vaccine compared to those who did not (AOR = 2.14, 95% CI: 1.86–2.45). Educational attainment showed a clear gradient: women with secondary education had 67% higher odds of awareness (AOR = 1.67, 95% CI: 1.38–2.02), and those with tertiary education had even greater odds (AOR = 2.14, 95% CI: 1.59–2.89), relative to women with no formal education.

Age was also a significant predictor. Compared to the 15–19 age group, women aged 35–39 had the highest odds of awareness (AOR = 1.76, 95% CI: 1.37–2.26), followed closely by those aged 30–34 (AOR = 1.69, 95% CI: 1.30–2.21). Marital status, ethnicity, and religious affiliation were also associated with awareness, though to a lesser extent. For example, women of Ewe ethnicity had slightly lower odds of awareness compared to Akan women (AOR = 0.82, 95% CI: 0.70–0.96). Region of residence had a substantial effect: women from Greater Accra (AOR = 2.01, 95% CI: 1.54–2.63) and the Upper East (AOR = 1.86, 95% CI: 1.42–2.44) had significantly higher odds of awareness compared to those in the Western region.

## 4 Discussion

This study investigated factors associated with malaria vaccine awareness among women aged 15–49 years in Ghana using the Demographic Health Survey data (DHS). We noted age, region of residence, educational level and religious affiliations influenced awareness of the malaria vaccine among the study population.

Our study reveals that internet use is the strongest predictor of malaria vaccine advertisement exposure among Ghanaian women. Women who reported internet use within the 12 months preceding the survey were significantly more likely to have encountered malaria vaccine advertisements. This aligns with evidence from Ghana, where the use of digital platforms such as WhatsApp by trained healthcare providers during the COVID‐19 pandemic led to measurable improvements in antenatal care attendance and health facility deliveries, suggesting that digital tools can enhance health system engagement and service uptake for malaria vaccine information [30]. Similarly, maternal health practitioners in Ghana have identified mobile health as a critical enabler of service efficiency and client education, particularly where internet‐supported tools facilitate remote consultations and the timely sharing of health information [31].

Despite these benefits, less than two‐thirds of women had used the internet in the past year, indicating a significant digital divide. This disparity is driven by infrastructural limitations, digital illiteracy, low device ownership and affordability challenges, particularly in rural and low‐income regions of Ghana [32, 33]. These patterns reflect broader concerns about digital redlining, where structural barriers prevent entire populations from benefiting equally from health innovations [34]. Women in socioeconomically disadvantaged settings are less likely to own smartphones or afford mobile data, making them disproportionately vulnerable to exclusion from internet‐based health campaigns. This raises important equity concerns as public health strategies increasingly rely on digital channels to disseminate vaccine‐related information. Without deliberate efforts to bridge digital gaps, particularly those shaped by gender and region, digital health campaigns may inadvertently deepen existing health inequalities in malaria vaccine exposure.

Education was another significant predictor of exposure to malaria vaccine advertisements. Women with higher levels of formal education were more likely to report exposure to promotional messaging. Education enhances cognitive skills and improves the ability to interpret health messages, making individuals more receptive to vaccine‐related information [35]. This is supported by Ghana's Free Compulsory Universal Basic Education policy, which has led to increased maternal health service utilisation and preventive behaviours such as antenatal care attendance [35]. Furthermore, education facilitates digital engagement, enabling women to access, evaluate and use online health content [36, 37, 38]. These findings underscore the role of education not only as a determinant of health literacy but also as a critical enabler of equitable vaccine information access.

Our results also show a modest increase in exposure to malaria vaccine advertisements with age. Women aged 35–39 were the most likely to report recent exposure, whereas those aged 15–24 were the least likely. These findings support prior research suggesting that younger women, particularly those who are out of school, face access challenges to maternal health services and may not view vaccination information as personally relevant [39, 40]. Social, economic and educational vulnerabilities at a young age limit engagement with the health system. Older women, on the other hand, tend to have more frequent interactions with maternal and child healthcare services, such as antenatal and postnatal care, which may increase their exposure to vaccine messaging [41]. This suggests that age may serve as a proxy for accumulated exposure to health services rather than being a direct determinant of malaria vaccine knowledge. Embedding vaccine messaging into routine maternal health contacts may help retain awareness among older women and improve outreach to younger and less‐engaged groups. However, regional disparities in access to health infrastructure and education may further influence exposure differences by age, particularly in rural and underserved regions [37].

Religious affiliation was also significantly associated with malaria vaccine advertisement exposure. Pentecostal and Christian women, particularly those living in urban and more educated settings, were more likely to report recent exposure compared to Muslim and traditionalist women. This reflects findings from Ghana that suggest religious identity shapes health behaviours by influencing exposure to health information, cultural norms and trust in biomedical systems. For instance, Immurana et al. found that Christian mothers were significantly more likely than their Muslim or traditionalist counterparts to accept malaria vaccination for their children, even after controlling for socioeconomic status [42]. Globally, similar trends have been documented, with children from Muslim households being more likely to be zero‐dose compared to Christian households, even after accounting for maternal education and household wealth [43]. These disparities highlight the potential for religious affiliation to serve as both a cultural and structural determinant of health exposure. Evidence from Ethiopia has shown that the inclusion of religious leaders in maternal health education campaigns can significantly increase antenatal care uptake, institutional deliveries and postnatal care [44]. These findings suggest that faith‐based strategies could be especially effective in closing exposure gaps in malaria vaccine campaigns in Ghana.

Regional residence also influenced the likelihood of exposure to malaria vaccine advertisements. Women living in the Western Region, used as the reference category, were more likely to report exposure than those in other regions. This may reflect stronger health communication infrastructure, media presence and antenatal care engagement in the Western Region. In contrast, regions such as the Northern, Upper East and Upper West have shown good performance on certain maternal health indicators like IPTp uptake but have not necessarily translated these gains into widespread awareness of newer interventions like the RTS, S malaria vaccine. These findings are consistent with Ghana's national health data, which show geographic disparities in health facility distribution, communication infrastructure and campaign penetration [33, 34]. Studies also indicate that low immunisation uptake in regions like Volta and the North may be due to structural and informational limitations rather than a lack of willingness [40, 41].

Other sociodemographic variables such as ethnicity and household headship were not significantly associated with exposure to malaria vaccine advertisements. This suggests that structural and informational access now outweighs traditional cultural hierarchies or household leadership in shaping exposure. This is consistent with Budu et al., who found that ethnic differences in child immunisation disappeared after adjusting for wealth, education and urban–rural residence [45]. Similarly, Santos et al. reported that religious affiliation and socioeconomic status were more predictive of zero‐dose vaccination than ethnicity across LMICs [43]. This may reflect a broader shift towards individual‐level access to information, particularly via mobile and community health platforms, as key determinants of vaccine exposure.

### 4.1 Recommendation

Although these recommendations stem from malaria vaccine exposure findings, they offer applicable insights for malaria vaccine awareness strategies, given the shared determinants of health communication and service uptake. The findings illustrate the power of varied communication approaches. Digital tools are still necessary in urban and connected communities, but non‐digital channels will remain crucial in rural and digitally disconnected communities. Approaches, including using local languages with radio, recalling (community drama), storytelling and home visits by health workers, can help bridge awareness gaps, leading to an encouraging outlook on malaria vaccine awareness. It is necessary to design differentiated approaches aimed at younger and adolescent women who are less willing to seek out maternal health services. Peer‐facilitated meetings, school‐based interventions and online and offline youth‐targeted health messages can also help to facilitate their involvement. For older women, active engagement must be encouraged by message reinforcement through antenatal and maternal services. The digital divide must be bridged as a matter of urgency. Investing in ICT infrastructure, mobile subsidies and programmes for digital literacy would further ensure the scale‐up of digital health innovations targeting the underserved. In addition to existing strategies, public health messaging should address regional disparities, engage faith‐based actors and focus on individual‐level access rather than household or ethnic identity. Multidisciplinary work between the health, education and communication sectors is vital so that nobody is left out.

### 4.2 Limitations

This study has several limitations that should be considered. First, the outcome variable, whether the respondent had heard of the malaria vaccine in the past 6 months, captures recent exposure to vaccine‐related information but does not assess the depth of knowledge, understanding or intent to vaccinate. Second, the use of self‐reported responses may introduce recall bias, particularly for questions involving media exposure or health service interactions. Third, due to data constraints, potentially relevant factors such as household income, partner influence and trust in the health system could not be included in the analysis, limiting a more comprehensive interpretation. Additionally, the DHS sampling design may underrepresent certain subgroups, such as out‐of‐school adolescents or women living in remote or underserved areas, who might have different levels of exposure to malaria vaccine messaging. Fourth, although internet use was used as a proxy for digital engagement, it does not provide detail on the type, frequency or quality of health‐related content encountered online. Finally, although the 2022 Ghana MIS is a high quality, nationally representative dataset, its timing may not reflect more recent developments in malaria vaccine awareness or rollout, particularly in a rapidly evolving public health landscape.

## 5 Conclusion

This study reveals that internet use, education, age, region and religion are key determinants of malaria vaccine awareness among Ghanaian women and likely predictors of malaria vaccine uptake. These findings underscore the growing influence of individual‐level access to health information, rather than traditional demographic roles such as household headship or ethnicity. Addressing systemic disparities in digital access, education and regional infrastructure is necessary for ensuring equitable malaria vaccine awareness and uptake and to have a meaningful impact on Ghana's 2028 Malaria Elimination Agenda and broader Sustainable Development Goals.

## Author Contributions

**Michael Sarfo**: conceptualization, methodology, data curation, formal analysis, validation, writing – review and editing. **Augustina Konadu Larbi‐Ampofo**: writing – original draft, writing – review and editing, formal analysis. **Olanrewaju Lawal**: writing – review and editing, writing – original draft, formal analysis. **Sandra Konadu Bonnah**: writing – original draft, writing – review and editing, formal analysis. **Gilbert Eshun**: conceptualization, writing – review and editing, supervision, formal analysis.

## Consent

The authors have nothing to report.

## Conflicts of Interest

The authors declare no conflicts of interest.

## Data Availability

The data that support the findings of this study are openly available in DHS at http://goo.gl/ny8T6X.

## APPENDIX A1

### A1.1

**Table:**

Variable	Variance inflation factor (VIF)	Tolerance indices (TI)
Age	1.062	0.942
Region	1.258	0.795
Religion	1.049	0.953
Education level	1.402	0.713
Ethnicity	1.026	0.975
Sex household health	1.050	0.953
Use of internet	1.215	0.823
